# Effect of Climate and Competition on Radial Growth of *Pinus sylvestris* var. *mongolica* Forest in Hulunbuir Sandy Land of Inner Mongolia, China

**DOI:** 10.3390/plants12132584

**Published:** 2023-07-07

**Authors:** Shuo Wen, Zhongjie Shi, Xiao Zhang, Leilei Pan, Semyung Kwon, Yuheng Li, Xiaohui Yang, Hanzhi Li

**Affiliations:** 1 Research Institute of Ecological Conservation and Restoration, Chinese Academy of Forestry, Beijing 100091, China; 2 Research Institute of Desertification Studies, Chinese Academy of Forestry, Beijing 100091, China; 3 Institute of Ecological Restoration, Kongju National University, Gongzhou City 32439, Republic of Korea

**Keywords:** competition, growth–climate correlation, tree-ring, linear mixed model, *Pinus sylvestris* var. *mongolica*

## Abstract

(1) Background: The forest of *Pinus sylvestris* var. *mongolica* is an important semi-arid ecosystem in Hulunbuir sandy land that plays a key role in the carbon cycle and wind erosion control. It is crucial to explore the main factors affecting the radial growth of trees of *P. sylvestris* var. *mongolica*. (2) Methods: The study established the tree-ring chronology of *P. sylvestris* var. *mongolica* and analyzed the relationships among the radial growth, competition index, and climate variables using correlation analysis and a linear mixed effect model to explore the influence of competition and climate on radial growth of *P. sylvestris* var. *mongolica*. (3) Results: The results indicated that tree growth is mainly affected by the maximum average temperature (T_max_) and precipitation in June and July of the current year and that tree growth significantly decreased with increasing competition pressure. Analysis of the linear mixed effect model showed that tree age, competition intensity, self-calibrating Palmer drought severity index (scPDSI) from May to July, and vapor pressure deficit (VPD) have a significant impact on radial growth. (4) Conclusions: The competition plays a dominant role in radial growth of *P. sylvestris* var. *mongolica* compared to climate factors. This study helps to understand the growth mechanism of *P. sylvestris* var. *mongolica* forests under climate change and provides a scientific basis for effective management of semi-arid forests.

## 1. Introduction

Climate variables are important factors affecting vegetation growth. Moisture condition is generally considered the key limiting factor affecting tree growth in arid and semi-arid areas of northern China [1]. Meanwhile, the continuous warming may lead to more frequent and stronger droughts, which further affect tree growth by altering the hydraulic traits of the wood. Many studies have been performed on the relationship between the radial growth of tree species (such as *Pinus massoniana*, *Larix gmelinii*, *Pinus tabulaeformis*, etc.) and temperature, precipitation, or drought in the semi-arid regions of northern China [2,3,4]. Wu et al. found that tree growth in inland arid Asia is mainly affected by the average temperatures in June and July, precipitation, or Palmer Drought Severity Index (*PDSI*) in April, June, and July [5]. In Hulunbuir sandy land, the radial growth of *Pinus sylvestris* var. *mongolica* natural forest is mainly affected by precipitation and drought during the growing season [6]. These studies mostly focused on the impact of climate variables on the growth of dominant trees in forests. However, the role of other factors in affecting tree growth was often neglected.

Considering the limited resources in arid and semi-arid areas of northern China, competition is also an important factor affecting tree growth. The influence of competition on the growth of trees is mainly manifested in the competition between trees and neighboring trees for space and resources such as moisture, nutrients, and light [7,8,9,10]. Stand density has a negative impact on the radial growth of trees during and after drought events [11]. Reducing tree competition by thinning to a certain degree can promote the growth of trees and enhance their adaptability to drought [12]. Competition will increase the sensitivity of trees to drought or precipitation, but it has little effect on the growth sensitivity to temperature [13,14,15]. The study also found that tree-to-tree competition can affect the response of *P. sylvestris* var. *mongolica* tree growth to climate [16] and enhances the ability of tree growth in response to climate [13]. Therefore, exploring the influence of tree-to-tree competition on radial growth is helpful to reveal the mechanism affecting tree growth and provides a basis for accurately evaluating and predicting the carbon sequestration and its dynamics in forests in the future.

With an in-depth understanding of the factors affecting the radial growth of trees, many studies are paying more attention to the comprehensive influence of climate and competition on the radial growth of trees [17,18,19]. Water, or heat, is the key factor restricting the growth of trees, but some studies found that competition plays a stronger role in determining the growth of trees than climate [18,20,21]. The role of competition and climate in influencing tree growth is still controversial and unclear. There are few studies on the influence of climate factors and competition on radial growth of *P. sylvestris* var. *mongolica* in the Hulunbuir area, and the limiting driver in determining the growth of trees in this area is still unclear.

*P. sylvestris* var. *mongolica* is a main tree species of afforestation in sandy land in northern China, with the characteristics of drought tolerance, cold tolerance, barren tolerance, and strong adaptability. The forest of *P. sylvestris* var. *mongolica* offers the important ecological services of windbreak and sand fixation, water conservation, and environmental improvement. The study selected *P. sylvestris* var. *mongolica* in Hulunbuir sandy land of Inner Mongolia, China as the target tree species, established tree-ring chronology, and explored the influence of competition and climate on the radial growth of trees using a linear mixed effect model and correlation analysis. It is helpful to reveal the mechanism in regulating tree growth, and this study provides a basis for scientific management and climate adaptation regulation in semi-arid sandy forests in the context of global change.

## 2. Results

### 2.1. Characteristics of Tree-Ring Chronology

**Figure 1:**
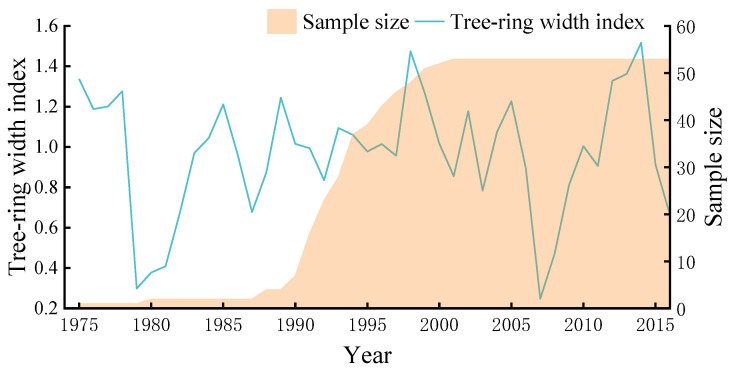
Tree-ring width index of *P. sylvestris* var. *mongolica* and its sample depths.

In this study, 106 cores from 53 trees were collected; the standard chronology of *P. sylvestris* var. *mongolica* is displayed in Figure 1. The statistical parameters of the standardized chronology of *P. sylvestris* var. *mongolica* stand are shown in Table 1. The time length of the standardized chronology is 42 years, ranging from 1975 to 2016. The mean sensitivity (MS) is 0.347, indicating that *P. sylvestris* var. *mongolica* is very sensitive to climate change in this region. The standard deviation (SD) is 0.391, indicating the chronology contains more climate signals. The signal-to-noise ratio (SNR) is 201.715, showing that the chronology contains a large amount of environmental information. The expressed population signal (EPS) is 0.995, which is much higher than the minimum standard of 0.85 [22], indicating the signals can represent the overall characteristics of the stand. The first-order autocorrelation coefficient (AC) is 0.598, which shows the growth of trees in the previous year has obvious influence on the growth of current year. The correlation coefficient (CC) for all series is 0.790, which indicates that the ring width varies consistently among different tree cores in the study area. Generally, the statistical characteristics show that the chronology of *P. sylvestris* var. *mongolica* is of high quality, meeting the requirements of this study.

### 2.2. Radial Growth–Climate Correlation

**Figure 2:**
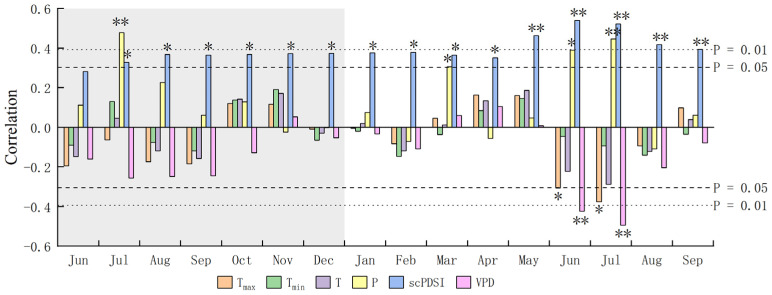
Correlation between ring-width chronology of *P. sylvestris* var. *mongolica* and monthly climatic variables. The shaded areas in the figure represent the data of last year. T_max_, monthly mean maximum air temperature; T_min_, monthly mean minimum air temperature; T, monthly mean air temperature; P, monthly precipitation; scPDSI, self-calibrating Palmer Drought Severity Index; and VPD, vapor pressure deficit. *, *p* < 0.05; **, *p* < 0.01.

Pearson correlation analysis was used to analyze the relationship between the chronology of *P. sylvestris* var. *mongolica* (1975–2016) and monthly climatic factors (Figure 2). The results showed that the radial growth of trees was negatively correlated with the average maximum temperature (T_max_) in June (r = −0.306, *p* < 0.05) and July of the current year (r = −0.375, *p* < 0.05) and positively correlated with the precipitation (P) in July (r = 0.478, *p* < 0.01) of the previous year and in March (r = 0.307, *p* < 0.05), June (r = 0.39, *p* < 0.05), and July (r = 0.446, *p* < 0.01) of the current year. It was significantly positively correlated with scPDSI in almost all months and significantly negatively correlated with VPD in June (r = −0.423, *p* < 0.01) and July (r = −0.494, *p* < 0.01) of the current year. Additionally, there is no significant correlation between radial growth of *P. sylvestris* var. *mongolica* trees and monthly average temperature (T) and average minimum temperature (T_min_).

### 2.3. Tree-to-Tree Competitions and Their Effect on Cumulative Basal Area Increment (BAI)

The competition index (CI) of *P. sylvestris* var. *mongolica* trees ranged from 1.36 to 10.94, with an average value of 3.73 and a standard deviation of 2.29, indicating a large difference in the competition pressure among the different trees of *P. sylvestris* var. *mongolica*. The cumulative BAI in the past 15 years ranged from 24.27 cm^2^ to 381.6 cm^2^, with an average value of 154.22 cm^2^ and a standard deviation of 87.92 cm^2^. The cumulative BAI is fitted to CI using a nonlinear model in different periods, and its marginal R^2^ is calculated. The results show that the cumulative BAI in the past 5, 10, and 15 years decreased in a power function with the increase in competition index, and every determination coefficient R^2^ was above 0.74, indicating the competition among trees significantly affected the growth of trees (Table 2).

### 2.4. Comprehensive Effect of Competition and Climate on Tree Growth

The linear mixed effect model analysis shows that tree age, CI, May–July scPDSI, and May–July VPD have obvious effects on the radial growth of *P. sylvestris* var. *mongolica* trees. The radial growth of trees increases with the increase in tree age and scPDSI but decreases with the increase in CI and VPD. The interaction between CI and May–July scPDSI also has an obvious influence on the radial growth of trees (Table 3). Considering the fixed effect (CI, tree age, scPDSI, and VPD) and random effect (tree variability), the model can explain 60.1% of variance. While excluding individual tree variability, fixed effect can explain 42.5% of variance (Table 3).

**Figure 3:**
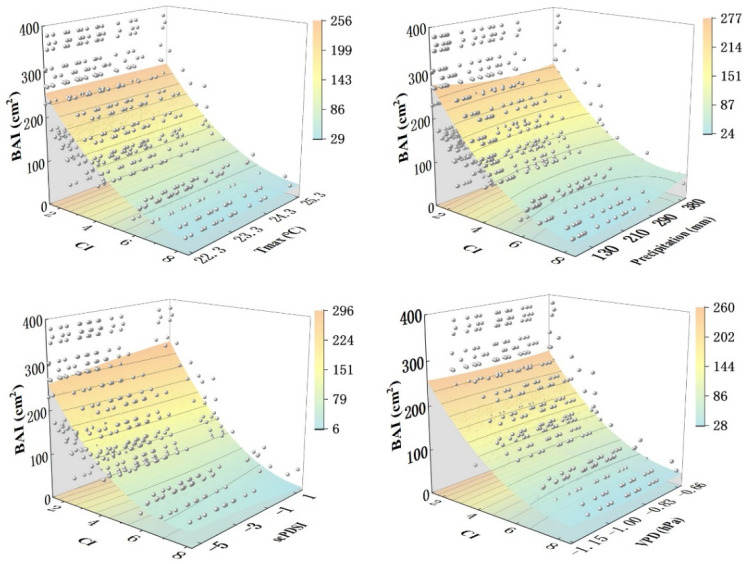
Relationship between cumulative BAI of *Pinus sylvestris* var. *mongolica* and climate variables and competition index.

The fitting results of BAI and CI and the climatic variables (T_max_, precipitation, scPDSI or VPD) are shown in Figure 3. The results show that CI plays a dominant role in regulating the radial growth of trees, while the role of climate variables is relatively small. When the climate factor is a certain value, cumulative BAI decreases with the increase in CI. When CI is a certain value, cumulative BAI is almost unaffected by T_max_. Cumulative BAI first decreases and then increases with the increase in precipitation. When CI is ≤5, cumulative BAI increases with the increase in PDSI and VPD. While CI is ≥5, cumulative BAI decreases with the increase in PDSI and first decreases and then increases with the increase in VPD.

## 3. Discussion

### 3.1. Effect of Climate on Tree Growth

Tree growth is influenced by both biological and environmental factors. Climate variables are the major environmental factors. This study shows that the radial growth of *P. sylvestris* var. *mongolica* in Hulunbuir sandy land is significantly affected by the average maximum temperature in June and July, a finding that is consistent with the findings of previous studies [16]. This may be because the high average temperature in June and July, coupled with the low precipitation in the Hulunbuir area, leads to soil moisture stress and stomatal closure, which inhibit the normal photosynthesis of *P. sylvestris* var. *mongolica* trees. However, some studies have shown a significant correlation between the tree growth of pine and the temperatures in October of the current or previous year, and differences in microclimate environments may explain the inconsistency of these results [23]. The Great Khingan Mountains are located in the transitional zone between the temperate and frigid zones, and the complex mountainous terrain results in significant differences in climate between the eastern and western sides. All in all, there is no doubt that temperature influences tree growth even though the results of different studies are inconsistent in different areas. In addition, in semi-arid ecosystems, temperature indirectly influences *P. sylvestris* var. *mongolica* individuals’ growth through evaporation, which removes soil moisture.

Our study found a significantly positive correlation (*p* < 0.05) between the radial growth of *P. sylvestris* var. *mongolica* and precipitation in March, June, and July of the current year, which is consistent with the findings of other studies in the region [24,25]. Kwon [16] also found that the radial growth of *P. sylvestris* var. *mongolica* was positively correlated with monthly precipitation in most months in Hulunbuir; the positive correlation was especially significant in September of the previous year and in May and July of the current year. However, the growth of *P. sylvestris* var. *mongolica* was significantly positively correlated with precipitation in December of the previous year or January of the current year in Mohe, but negatively correlated in February of the current year and positively correlated in June of the current year [26]. The results also indicate the time lag effect of precipitation on the radial growth of trees. The precipitation in autumn or winter of the previous year can enhance soil moisture, making it less likely for tree growth to be limited by water availability during the early stages of the growing season.

The scPDSI, an indicator that considers the comprehensive effect of precipitation and temperature, is an important indicator for assessing the availability of water resources for plant growth [27]. Our study found a significant correlation between the radial growth of *P. sylvestris* var. *mongolica* and the scPDSI from July of the previous year to September of the current year, especially from May to September of the current year. This may be due to higher evapotranspiration than precipitation in the Hulunbuir area, which makes water availability a critical limiting factor for tree growth [6].

### 3.2. Effect of Competition on Tree Growth

Competition is also an important factor affecting the radial growth of trees. Our study found a power function relationship between the cumulative BAI and competition index (competition pressure), where radial growth decreased with the increase in competition pressure, which is consistent with previous studies [13,18,19]. Kang et al. [28] found that the fitting effect between the growth of *Pinus sibirica* in the Altai Mountains of Xinjiang, China and the competition index had been good for the past 15–30 years, with the best fitting effect about 30 years ago. The growth of *Pinus massoniana* in the subtropical region of China also decreases with increasing competition index [29]. In the broad-leaved Korean pine forest of the Liangshui Nature Reserve of China, the radial growth of large diameter-at-breast-height (DBH) trees was mainly affected by topographical factors, while the radial growth in small-DBH trees was mainly controlled by competition [30].

Many studies have found that reducing competition pressure by thinning can promote tree growth [31,32] or alleviate growth decline [33]. A study of a broad-leaved Korean pine forest in Jilin, China showed that the trees retained after thinning had higher growth rates and lower growth decline caused by climate warming and drying. In particular, heavy thinning increased the recovery resilience of the trees after extreme drought [34]. Kang et al. [35] found that competition exacerbates the sensitivity of tree growth of *Pinus koraiensis* to precipitation in June but has no significant effect on the relationship between tree growth and temperature in the Lesser Khingan Mountains. These findings suggest that competition is an important limiting factor for tree growth of forests, so it is necessary to consider the influence of competition when analyzing the relationship between tree growth and environmental factors. Additionally, the impact of competition on tree growth may vary over time due to other disturbances, such as wildfire, tree death, etc., indicating the importance of long-term monitoring and management of forest ecosystems to optimize forest health and productivity.

### 3.3. Comprehensive Effects of Climate and Competition on Tree Growth

Tree growth is jointly controlled by the tree-to-tree competition and climatic variables. Climatic factors mainly affect the radial growth of trees by regulating their physiological activities, while competition mainly affects radial growth by controlling the resource utilization of trees, such as trees’ utilization of water, nutrients, light, space, etc. Both factors have a considerable impact on tree growth [17]. At the same time, their interaction also jointly affects tree growth [18,36].

In this study, we found that climate and competition jointly affect the radial growth of *P. sylvestris* var. *mongolica* in Hulunbuir sandy land with competition playing a dominant role. Many studies have also suggested that competition, rather than climate, is the primary limiting factor for tree growth [29,37]. For example, Zhang et al. [38] found that competition, rather than climate, is the main factor causing growth decline of trees in boreal forests in western Canada. Competition also plays a dominant role in regulating the radial growth of *Pinus massoniana* in subtropical China [29].

Nicklen et al. [36] also found that competition and stand water characteristics mitigated the response of *Picea glauca* in Denali National Park, Alaska to climate change, jointly regulating tree growth. Additionally, competition affects tree growth through its interaction with climate variables, further regulating the response of tree growth to drought. Our study found that the interaction between competition and drought significantly affects the radial growth of *P. sylvestris* var. *mongolica* in Hulunbuir sandy land. Therefore, forest cultivation should be emphasized in the management of *P. sylvestris* var. *mongolica* forests, and reasonable measures such as thinning should be taken to regulate stand density and optimize tree-to-tree competition, enhancing the resistance to drought of the forest and effectively improving stand productivity, promoting sustainable forest development.

## 4. Materials and Methods

### 4.1. Study Area

**Figure 4:**
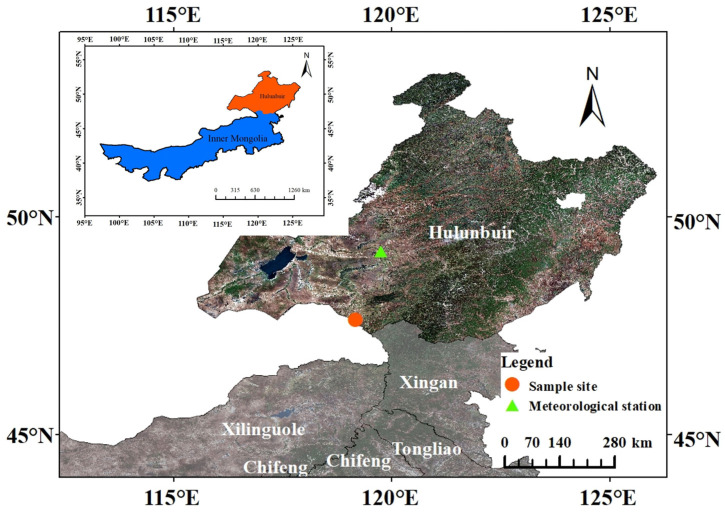
Location of sample site and meteorological station.

The study area is located in Handagai Sumu (47°38′ N, 119°10′ E) in Xinbarhu Zuoqi, Hulunbuir, Inner Mongolia, China (Figure 4). It is a typical semi-arid temperate continental climate with a long winter and warm but short summer, abundant sunshine, and large diurnal temperature range. The frost-free period is from 90 to 100 days, the annual average temperature is about 0.2 °C, the annual precipitation ranged from 280 mm to 400 mm, and the annual potential evaporation ranged from 1400 mm to 1990 mm. The soil is a type of aeolian sandy soil, with loose structure and low moisture. It belongs to the forest–grassland ecotone in the landscape. The landforms are mostly fixed or semi-fixed sand dunes with northwest-southeast directions and a relative height of 10–30 m. The vegetation is mainly natural pure forest of *P. sylvestris* var. *mongolica* and grassland, accompanied by species such as *Betula platyphylla*, *Populus davidiana*, *Corylus heterophylla*, *Rosa davurica*, etc. The understory vegetation is dominated by xerophytes, including *Filifolium sibiricum*, *Stipa grandis*, *Leymus chinensis*, *Sanguisorba officinalis*, etc.

### 4.2. Climatic Data

The climate data used in this study are from the Hailar National Meteorological Station of the China Meteorological Data Network, http://data.cma.cn/site/index.html (accessed on 22 December 2020) (49°13′ N, 119°45′ E, and 610.2 m above sea level). Climatic variables include monthly average temperature (T), monthly average minimum temperature (T_min_), monthly average maximum temperature (T_max_), monthly precipitation (P), and saturated water vapor pressure difference (VPD). The drought is characterized by the self-correcting Palmer drought severity index (scPDSI), which comes from the PDSI grid (47.5° N, 119° E in this study) data of the Royal Dutch Meteorological Institute, http://climexp.knmi.nl/ (accessed on 2 February 2023). The time period is from 1958 to 2017. In addition to the climatic variables of the current year, the climate variables from June to December of the previous year are also used in the analysis.

### 4.3. Field Investigation and Establishment of Tree-Ring Chronology

**Figure 5:**
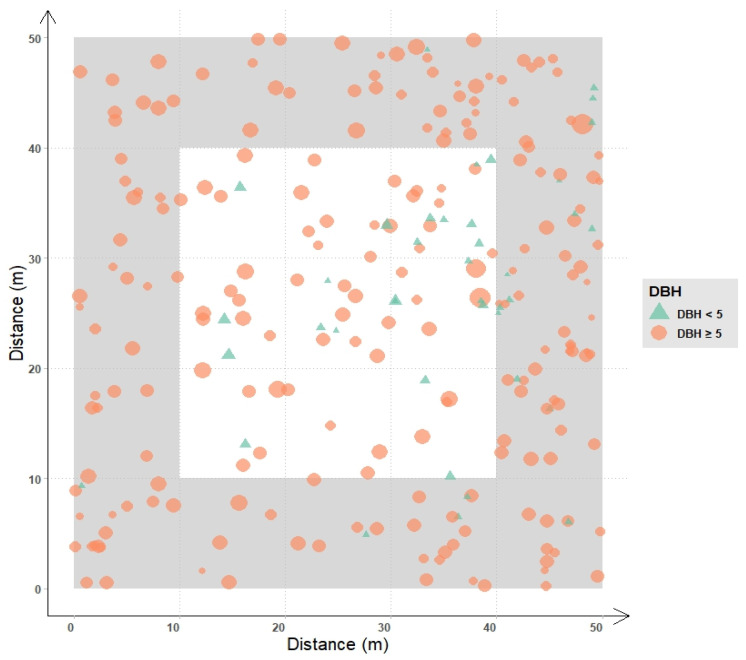
Sample Trees Distribution with Different DBH. The symbol size is proportional to DBH. Circles represent trees with DBH ≥ 5 cm, and triangles represent trees with DBH ≤ 5 cm.

A 50 × 50 m plot was developed in the natural stand of *P. sylvestris* var. *mongolica* without obvious disturbance or damage in Hulunbuir sandy land, and the information of each tree was recorded, including DBH, height, crown width, height under branches, and location. All trees with DBH ≥ 5 cm and tree height ≥ 2 m within the range of 30 × 30 m in the center of the plot were sampled, and at least 2 cores were sampled from each tree. For the non-sampled young trees, age was estimated by counting the branching layers (Figure 5).

The tree-ring cores were firstly fixed, dried, and polished, and the ring width was measured using LINTAB 6 with an accuracy of 0.01 mm. Cross dating was performed using the skeleton graph method and tested using the COFECHA program [39] to remove unreasonable sample data. Standardized chronology (STD) was established using the dplR” package in R software [40] to eliminate tree growth trends [41]. The parameters were calculated, including mean sensitivity (MS), signal-to-noise ratio (SNR), expressed population signals (EPS), etc.

Additionally, the BAI was established considering the DBH and skin thickness of each tree by “dplR” package, and the cumulative BAI of each target tree in the past 5, 10, and 15 years was calculated [42]. The formula of BAI is as follows:

(1) $$\mathrm{BAI}=\pi \left(R_{t}^{2}-R_{t-1}^{2}\right)$$

where R_t_ and R_t−1_ are radius at breast height of trees corresponding to years t and t − 1, respectively (cm).

### 4.4. Competition Index (CI)

Competition is the interaction between organisms in the utilization of space and resources, which will limit the growth of trees [43]. Many models have been proposed to characterize the competition intensity of trees, including distance-dependent [44,45] and distance-independent [46,47] models. This study adopted the Hegyi competition model [48], which is easy to calculate and widely used, and its formula is as follows:

(2) $$\mathrm{CI}=\sum_{i=1}^{N}\frac{D_{j}}{D_{i}}\times \frac{1}{L_{\mathrm{ij}}}$$

where  CI is the competition index of target tree i, $D_{i}$ is the DBH of the target tree i, $D_{j}$ is the DBH of the competing tree j, $L_{\mathrm{ij}}$ is the distance between target tree i and competing tree j, and N is the number of competing trees. According to previous research [49], trees with DBH ≥ 5 cm within a radius of 8 m are used as competing trees in this study. Since trees will grow with time, their competitive indexes will change, but for the target tree, the relative competitive pressure is constant. Therefore, the temporal change in competitive pressure is ignored in this study. The current competitive index is used to simulate the growth of the target tree in different periods.

### 4.5. Statistical Analysis

Pearson correlation coefficients between the standardized chronology and climate factors were calculated by “treeclim” package in R software, and significance tests were performed using the bootstrap resampling. A linear mixed effect model is an extension of a linear model that includes fixed effects and random effects. This model does not require the independence and homogeneity of variance of the dependent variable, making it more widely applicable. In this study, a linear mixed effect model was used to analyze the effects of competition and climate on the radial growth of trees. The BAI of each tree was used as the dependent variable in different years. Climatic variables, such as precipitation, average temperature, scPDSI, and VPD, along with competition index and tree age, were used as fixed effects. Additionally, the interaction between competition index and climate factors and age were considered, while individual trees were treated as random effects. The fixed effect model is shown as follows:

(3) BAI=a+b×CI+c×Age+d×Clim+e×CI×Clim+ε

where CI is the competition index, Age is the tree age, Clim represents the climatic variables, and  CI×Clim represents the interaction between CI and the climatic variables. a~e are independent variable coefficients; ε is a random effect, and BAI is the basal area increment. The linear mixed effect model is fitted with the “lme4” package in R software, and the marginal R^2^ and conditional R^2^ are calculated with the “MuMIn” package [50] to evaluate the model quality. Nonlinear surface analysis was performed by Origin 2021 software to fit the effect of the competition index and climate variables to annual radial growth.

## 5. Conclusions

This paper explores the effects of climate and competition on the radial growth of *P. sylvestris* var. *mongolica* in Hulunbuir sandy land. The results show that tree growth is mainly affected by the average maximum temperature and precipitation in June and July and that it significantly decreased with increasing competition pressure. Linear mixed effect model analysis revealed that tree age, CI, May–July scPDSI, and May–July VPD have a significant impact on radial growth. Although competition and climate jointly affect the radial growth of trees, competition is the primary factor affecting the radial growth of *P. sylvestris* var. *mongolica* in the study area. The study is helpful to understand the main influencing factors of tree growth in Hulunbuir sandy land and to provide a scientific basis for determining reasonable forest management in the region.

## Figures and Tables

**Table 1:** Statistics parameters of tree-ring standard chronology of *P. sylvestris* var. *mongolica* in Hulunbuir sandy land.

Period	Trees/Cores	MS	SD	CC	SNR	EPS	AC
1975–2016	53/106	0.347	0.391	0.790	201.715	0.995	0.598

MS, mean sensitivity; SD, standard deviation; CC, correlation coefficient for all series; SNR, signal-to-noise ratio; EPS, expressed population signal; and AC, first-order autocorrelation coefficient.

**Table 2:** Nonlinear modeling (y = ax^−b^) parameters between cumulative BAI and CI of *P. sylvestris* var. *mongolica* forest in Hulunbuir sandy land.

Cumulated Time Interval (Year)	a	b	Marginal R^2^
5	165.35	0.990	0.7446
10	265.41	1.005	0.7602
15	434.52	1.056	0.7633

In the table, a and b indicate the parameter of the models.

**Table 3:** Effects of tree age, CI, scPDSI, and VPD on the growth of *P. sylvestris* var. *mongolica* based on linear mixed effect model.

	Predictor Variable	Estimated Value	Range	*p*
**Fixed effect**	Inception	1805.67	1545.71–2065.63	<0.001
	Tree age	32.73	29.07–36.39	<0.001
	CI	−197.22	−238.56–−155.87	<0.001
	May–July scPDSI	110.95	84.01–137.89	<0.001
	May–July VPD	−725.95	−982.46–−469.44	<0.001
	CI × May–July scPDSI	−16.57	−22.56–−10.57	<0.001
**Random effects**	σ^2^	217,954.60		
	τ_00 TreeID_	96,515.89		
	ICC	0.31		
	N_TreeID_	53		
	sample size	1267		
	Marginal R^2^/Conditional R^2^	0.425/0.601		

In the table, σ^2^ is the variance of residuals, τ is the variance caused by random effects, ICC is intra-class correlation coefficient, N is the number of plots, Marginal R^2^ is the coefficient of variation for fixed effects, and Conditional R^2^ is the coefficient of variation for fixed and random effects.

## Data Availability

All data are presented in the main text.
